# Fabrication of Mechanically Stable Superhydrophobic Aluminium Surface with Excellent Self-Cleaning and Anti-Fogging Properties

**DOI:** 10.3390/biomimetics2010002

**Published:** 2017-02-23

**Authors:** Priya Varshney, Soumya S. Mohapatra, Aditya Kumar

**Affiliations:** Department of Chemical Engineering, National Institute of Technology Rourkela, Odisha 769008, India; 514CH1002@nitrkl.ac.in (P.V.); mohapatras@nitrkl.ac.in (S.S.M.)

**Keywords:** superhydrophobic, water-repellent, chemical etching, self-cleaning, anti-fogging

## Abstract

The development of a self-cleaning and anti-fogging superhydrophobic coating for aluminium surfaces that is durable in aggressive conditions has raised tremendous interest in materials science. In this work, a superhydrophobic Al surface was synthesized by employing chemical etching technique with a mixture of hydrochloric and nitric acids, followed by passivation with lauric acid. The surface morphology analysis revealed the presence of rough microstructures on the coated Al surface. Superhydrophobicity with water contact angle of 170 ± 3.9° and sliding angle of 4 ± 0.5° was achieved. The surface bounced off the high-speed water jet, indicating the excellent water-repellent nature of the coating. It also continuously floated on a water surface for four weeks, showing its excellent buoyancy. Additionally, the coating maintained its superhydrophobicity after undergoing 100 cycles of adhesive tape peeling test. Its superhydrophobic nature withstood 90° and 180° bending and repeated folding and de-folding. The coating exhibits an excellent self-cleaning property. In a low temperature condensation test, almost no accumulation of water drops on the surface showed the excellent anti-fogging property of the coating. This approach can be applied to any size and shape of Al surface, and hence has great industrial applications.

## 1. Introduction

The wettability of a solid surface as characterized by contact angle and sliding angle is determined by the combined effect of the chemical composition and the surface morphology [1,2]. Superhydrophobic surfaces show water contact angle greater than 150° and sliding angle smaller than 10°. These surfaces are bioinspired from natural sources such as lotus and rice leaves, and butterfly wings, due to the presence of micro- or nanostructures on their surfaces [3,4]. Recently, these surfaces have shown great interest due to their self-cleaning [5], anti-fogging [6], anti-reflective [7], and microfluidic [8] properties. Many novel methods for the fabrication of these biomimetic surfaces have been developed, such as anodic oxidation [9,10], chemical deposition [11], chemical etching [12,13,14,15], chemical vapor deposition [16,17,18,19], colloidal self-assembly [20,21,22], electrospinning [23,24], sol–gel [25,26] and some others [27,28].

Aluminium has plenty of applications in industry as well as in household activities thanks to its light weight, excellent heat and electrical conductivities, natural availability, and high mechanical properties [29,30], but these applications are limited due to the corrosion or deterioration of Al. A protective superhydrophobic layer on the Al surface repels the water, dirt, and moisture from the surface. This results in a dry and clean surface, and it slows down the corrosion or deterioration process. It is therefore highly desirable to create a biomimetic Al surface which is superhydrophobic in nature with self-cleaning, anti-corrosive, anti-fogging properties [31].

Superhydrophobic coatings can be fabricated by using the above-mentioned synthesis techniques. Some of them are simple and inexpensive, whereas some require special reagents and equipment, leading to a costly coating. Among them, chemical etching is a facile method to prepare superhydrophobic coating for Al substrate, as it has dislocations on its surface and selective dislocation etching can be easily done. Additionally, chemical etching also increases its anti-corrosive property [32]. Recently, several studies on the creation of superhydrophobic coatings on Al surfaces using chemical etching technique have been done. For instance, He et al. [33] created roughness on Al surfaces by chemical etching using boiling water and then achieved superhydrophobicity by treating roughed Al with polyethylenimine (PEI) and STA. Ren et al. [34] prepared superhydrophobic Al surfaces by creating roughness with hot water and then dipping the roughed Al in fluorosilane solution. Guo et al. [35] achieved superhydrophobic Al surfaces by roughening the Al surface by immersing in sodium hydroxide solution and then treating with fluorinated silane. Saleema et al. [36,37] obtained a superhydrophobic Al surface by treating it with a mixture of fluoroalkylsilane and sodium hydroxide solution. Xie et al. [38] generated roughness on an Al surface by sodium hydroxide etchant and then created superhydrophobicity by immersion of the rough Al in lauric acid solution. Fu et al. [39] fabricated superhydrophobic Al surfaces by chemical etching using mixed Cu(NO_3_)_2_ and HNO_3_ etchant solution followed by silane coating. Wang et al. [40] created roughness on an Al surface using an HNO_3_ and H_2_O_2_ mixed etchant solution and then treated the roughened Al in a mixed solution of stearic acid and *N*,*N*-dicyclohexylcarbodiimide to achieve superhydrophobicity. Qian et al. [13] prepared a superhydrophobic Al surface using Beck’s dislocation etchant and fluorination. A superhydrophobic Al surface was achieved by chemical etching with sodium hydroxide etchant followed by fluorosilane coating [41]. Li et al. [42] used hydrochloric acid as chemical etching solution to prepare a superhydrophobic surface on Al alloy. Superhydrophobic Al surfaces were prepared by chemical etching and anodization using hydrochloric acid, sulphuric acid, and boracic acid followed by self-assembly of fluoroalkylsilane. Zhang et al. [43] obtained superhydrophobic Al surfaces by immersing in hydrochloric acid and myristic acid solution. 

Despite having excellent properties (e.g., self-cleaning, anti-corrosive, anti-icing, etc.), superhydrophobic surfaces are not widely industry applicable because of a lack of mechanical stability. In the current work, a bioinspired superhydrophobic coating on an Al surface was prepared by chemical etching technique using a mixture of hydrochloric and nitric acid etchant and coating with lauric acid solution. Additionally, the wetting stability of the coating under mechanical disturbances was studied. Further, self-cleaning and anti-fogging characteristics of the coating were also studied.

## 2. Materials and Methods

### 2.1. Materials

Aluminium sheets (A110, >99% purity, size 7 cm × 2 cm × 1 mm, and 1 g (Indofoil Pvt. Ltd., Kosabadi, Chattisgarh, India) were used as substrates for developing the superhydrophobic coatings. Nitric acid (Emplura, Merck Specialties, Pvt. Ltd., Bangalore, Karnataka, India), hydrochloric acid (35%, Emplura, Merck Specialties, Pvt. Ltd.), ethanol (Emsure, Merck KGaA, Darmstadt, Germany), and lauric acid (99%, Loba Chemie Pvt. Ltd., Mumbai, Maharashtra, India) were used for preparation of superhydrophobic coatings.

### 2.2. Synthesis of Superhydrophobic Coatings

**Figure 1:**
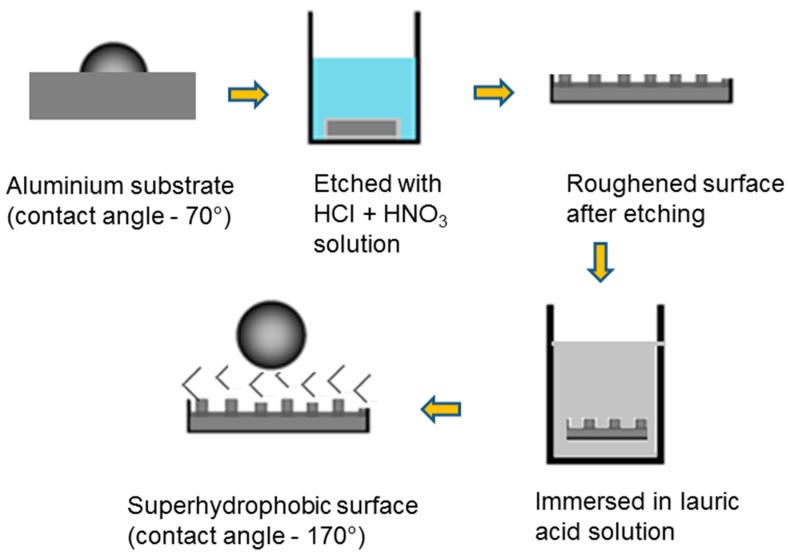
Schematic diagram of coating preparation by a two-step process: chemical etching with a mixture of hydrochloric and nitric acids followed by passivation with lauric acid.

Synthesis of the superhydrophobic coating on the Al surface includes two steps: first, creation of a rough Al surface, and then lowering the surface energy of the roughed Al surface (Figure 1). The Al substrate was initially cleaned with acetone and distilled water three times, then the Al substrate was immersed in a five times diluted solution of a mixture of HNO_3_ and HCl (ratio 1:3) in distilled water solution for 30 min. The acidic solution roughed the Al surface. Subsequently, the Al substrate was first rinsed with distilled water for 1 min, and then with ethanol. After etching, the sample was kept in a hot air oven at 60 °C for one hour. Afterwards, the etched Al sample was immersed in 20 g/L ethanol solution of lauric acid for 24 h, and then it was dried in air for 24 h. Lauric acid lowered the surface energy of the Al surface. The experiment was performed under atmospheric conditions.

### 2.3. Characterization of Superhydrophobic Coatings

Contact angle measurements were done by using a drop shape analyzer (DSA25, Krüss GmbH, Hamburg, Germany) with a droplet of distilled water having a drop volume range of 4–5 μL. The experiments were repeated at five different points on each sample, and their average with standard deviation was calculated. Surface morphologies of uncoated and coated samples were examined using scanning electron microscopy (SEM; Nova NanoSEM, FEI, Hillsboro, OR, USA). The roughness of the uncoated and coated samples was measured by a stylus surface profilometer (Dektak 150, Veeco Instruments Inc., Plainview, NY, USA). Five scans of 1.5 mm were carried out on different surface positions of each sample in order to drive the corresponding roughness.

In floatation on water surface test, a coated sample was kept floating on the water’s surface in a petri dish, and floatation time was recorded until the sample started sinking. 

A water jet impact test was carried out by spraying water on uncoated and coated samples from a 25 mL syringe. The water jet was kept about 3 cm above the surface with an angle of nearly 45° for 1 min along with an impact speed of 2.6 m/s. The interaction between the water jet and surface was later observed.

Mechanical durability of the superhydrophobic Al surface was tested by adhesive tape peeling and surface bending tests. In surface bending tests, coated samples were bent in different directions and angles (90° and 135° with radii of curvature of about 0.05 and 0.25 cm), and folded and de-folded multiple times. To check superhydrophobicity, water droplets were placed at different positions on bending or kink areas. For adhesive tape peeling test, a 3 M standard electrical insulation tape of adhesive strength of 100 N/m (Paramount Adhesive Pvt. Ltd., Valsad, Gujarat, India) was glued and unglued multiple times on the coating. Peeling tests were continued until the coating lost its superhydrophobicity.

The self-cleaning test was performed by sprinkling 0.5 g chalk powder of particle size of 4.5 µm on the uncoated and coated Al surfaces. Water droplets were slowly dropped on the chalk powder-sprinkled surfaces and the flow of droplets was observed. In the anti-fogging test, uncoated and coated Al samples were kept in the deep freezer (−18 °C) for five hours, and then they were kept in humid atmosphere of 80% relative humidity.

## 3. Results and Discussion

### 3.1. Surface Morphology and Wetting Properties

Prior to the synthesis of the coating, the Al substrate was cleaned and the contact angle was measured. It was found that the untreated Al surface had a water contact angle of 70 ± 4.5°, and this means that it is hydrophilic in nature. The superhydrophobic coating on the Al substrate was synthesized by a two-step process: roughness on the Al surface was generated by etching with HNO_3_ + HCl acidic solution, and then the surface energy of the roughed Al substrate was lowered by immersion into an aqueous ethanol solution of lauric acid. Water contact angle and surface morphology of the modified Al surface were characterized using contact angle measurement technique and SEM, respectively. 

Surface modification with lauric acid results in the formation of a sponge-like layer on the etched Al surface due to the presence of a carboxyl group that reacts with the Al atom through a dehydration process:

Al^3+^ + 3CH_3_(CH_2_)_10_COO^−^ → Al(CH_3_(CH_2_)_10_COO)_3_

Bonding of the long non-positive end of the alkyl to the etched Al surface creates a low energy surface. This increases the water contact angle. Water static and sliding contact angles were found to be 170 ± 3.9° and 4 ± 0.5°, respectively.

**Figure 2:**
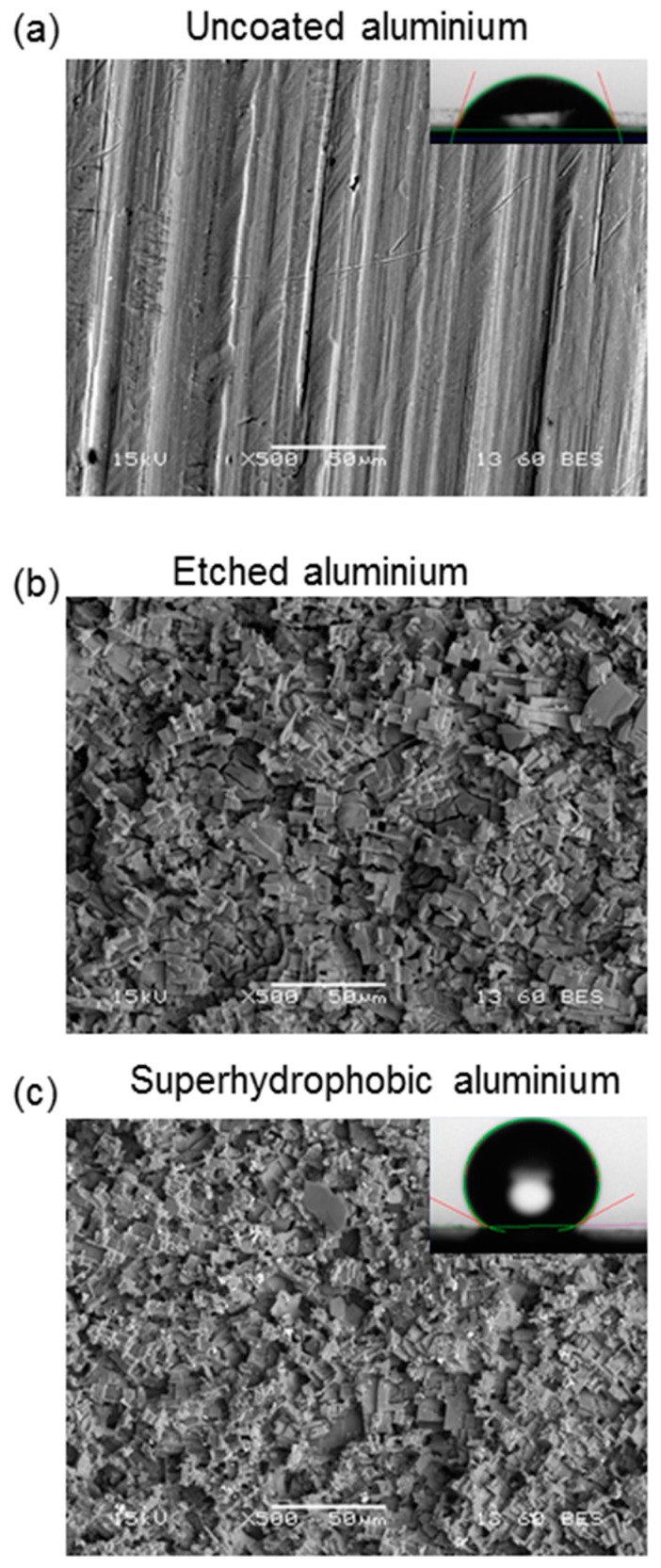
Scanning electron microscopy (SEM) images of (**a**) uncoated, (**b**) etched, and (**c**) superhydrophobic Al surfaces. Inset shows a water drop image on corresponding Al surfaces.

Figure 2 shows the SEM images of untreated and treated Al samples. When Al was etched in acidic solution, surface morphologies changed and micro-pits formed. Roughness of the untreated and coated surfaces was measured, and the average surface roughness of the superhydrophobic Al was found to be 8.76 ± 1.50 µm, while the average surface roughness of the untreated Al was observed as 0.58 ± 0.22 µm. After immersion into lauric acid solution, aluminium laurate (Al(CH_3_(CH_2_)_10_COO)_3_) forms with hydrophobic tails on the rough Al surface, which promotes water repellency on the originally hydrophilic Al. Air is likely to be present in micropits or microgrooves on the rough treated Al surface, and this trapped air may further increase the water contact angle [2].

### 3.2. Wetting Stability of the Coatings under Perturbation Conditions

**Figure 3:**
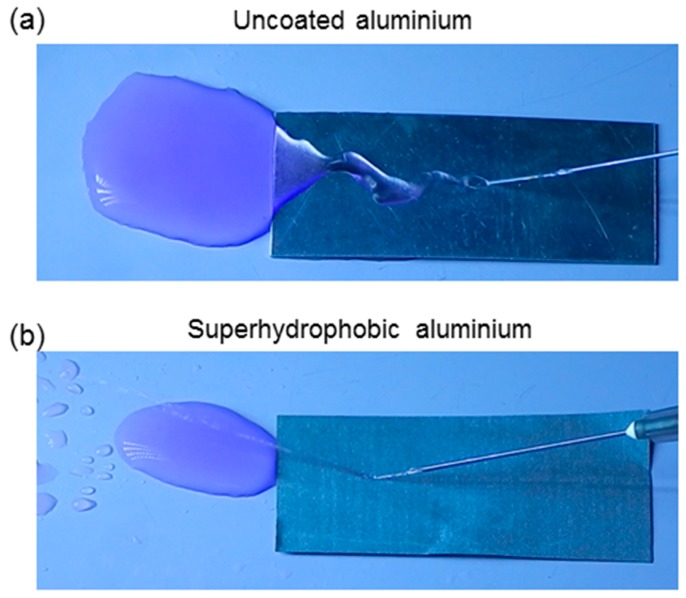
Optical images of water jet impact on (**a**) uncoated and (**b**) superhydrophobic Al surfaces.

To check the mechanical durability of the superhydrophobic Al, water jet impact, adhesive tape peeling, and surface bending tests were carried out. Figure 3 shows the water jet impact test for both uncoated and coated Al surfaces. It reveals that the untreated Al surface does not prevent the water from spreading on its surface, and water spreads immediately without bouncing off. This is due to the smoothness of the untreated Al sample with its hydrophilic nature. Whereas the superhydrophobic Al surface prevents water from spreading, and it bounces off the water jet in the opposite direction, as shown in Figure 3. This is because of the superhydrophobic nature of the coating. The presence of air pockets and the lower surface energy on the surface may not allow the impacting water jet to enter into the rough structure of the surface, and it leads to the jet bouncing off from the surface [44,45]. Generally, an impacting water stream can irreversibly ruin the water-repellent properties of the superhydrophobic surface [46,47,48]. The water jet was targeted at the same position for five minutes, and the water jet was still continuously bouncing off the superhydrophobic surface, indicating excellent water-repellent and mechanical strength of the coating.

**Figure 4:**
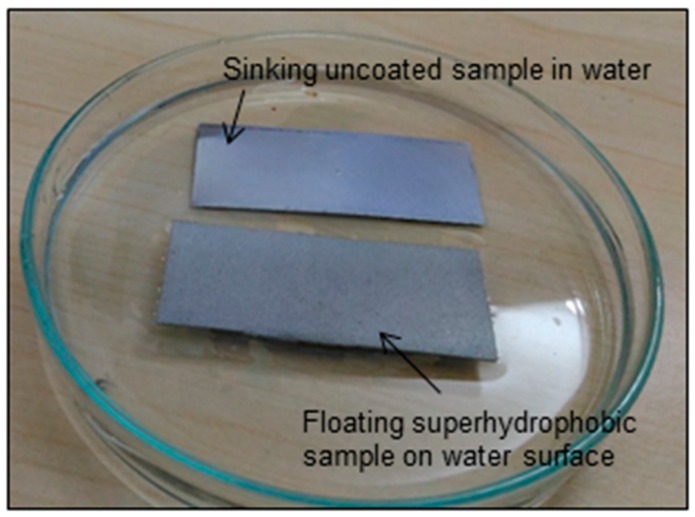
Optical images of the superhydrophobic Al sample floating on the water’s surface and uncoated Al sinking in water.

Figure 4 shows the floating uncoated and coated samples on the water surface. The uncoated Al sample was not able to float, and sank immediately in the water. On the other hand, the coated sample did not sink and started floating on the water’s surface. The superhydrophobic Al appears to repel the water, and the weight of the displaced water becomes more than the body of the sample, remaining on the water’s surface for four weeks until it was taken out. This indicates the excellent water-repellent nature of the coating. 

**Figure 5:**
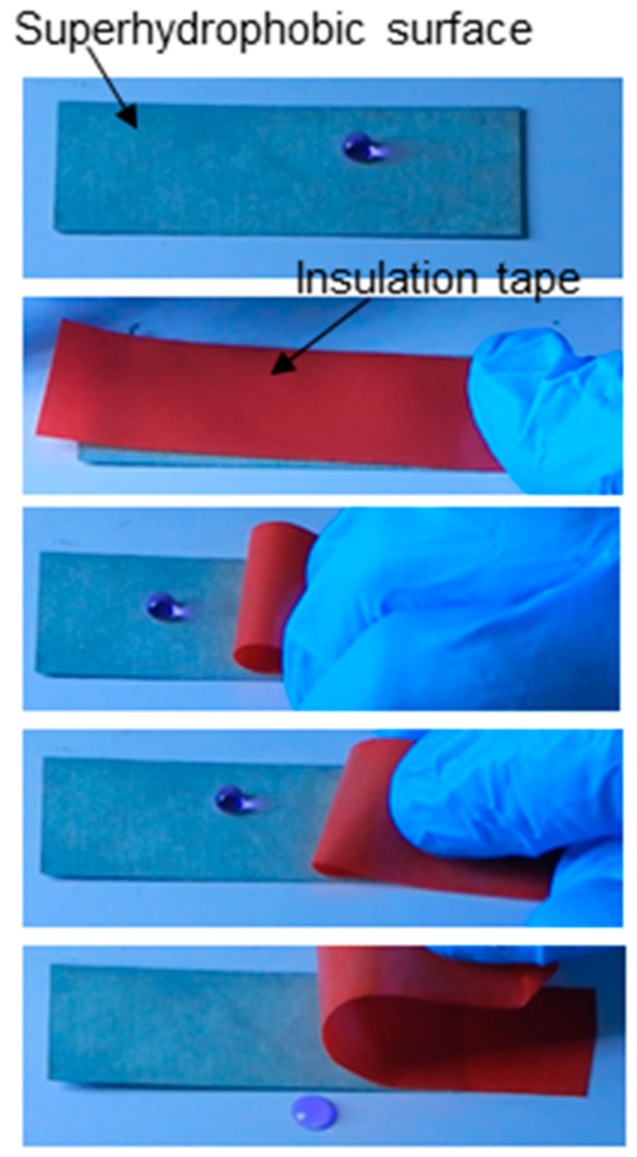
Optical images of different stages of adhesive tape peeling test.

To check adhesive strength of the coating, an electrical insulation tape was glued and unglued multiple times on the coated Al surface. Figure 5 shows the different stages of the peeling test. It is observed that the coating remained unaffected for up to 100 cycles of peeling, as the water droplets fall off the surface. After 100 cycles, the coating lost its superhydrophobicity and achieved sticky superhydrophobicity. In this situation, the static contact angle decreased and the sliding angle rapidly increased and the water drop could not roll down and remained stuck on the surface [49]. Water droplets did not even fall off the surface when the surface was tilted at 90°. This is because the coating surface was destroyed by multiple gluing and de-gluings of tape on the surface. Recently, Wang et al. [50] reported a mechanically-stable superhydrophobic steel surface which endured its surface microstructure against 70 times adhesive tape peeling tests.

**Figure 6:**
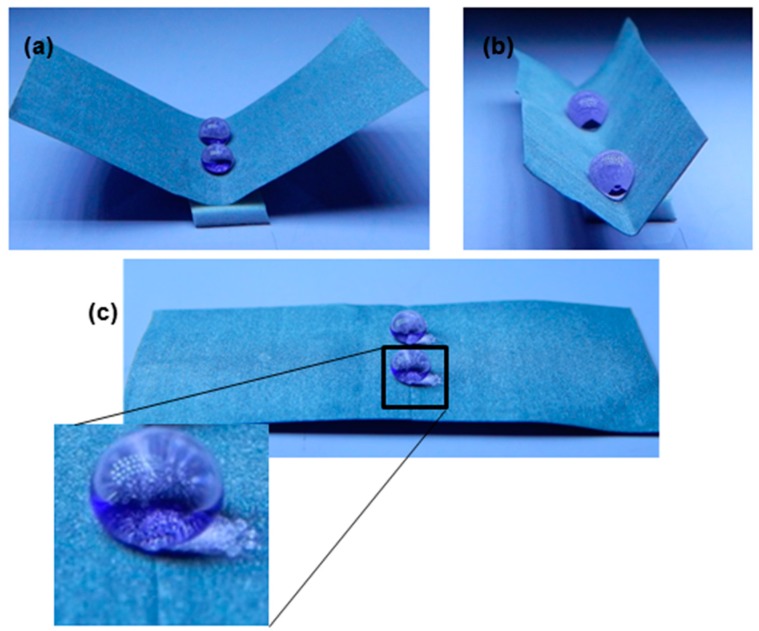
(**a**,**b**) Optical images of water droplets on a bendable (about 90°) superhydrophobic Al surface; (**c**) Optical image of water droplets on the superhydrophobic Al surface after bending at 180° and being released back to the original position.

By introducing mechanical disturbances such as surface bending and folding, the wettability of the superhydrophobic surface is affected [51,52]. Therefore, the effect of bending and folding on the superhydrophobicity of the present coated surface was also studied. In this regard, the superhydrophobic sample was bent in different directions and angles. It was also repeatedly folded and de-folded. Bending and folding do not exhibit any effect on the superhydrophobic properties of the coated Al. Figure 6 shows how the water droplets form a bead-like shape at 90° and 180° bending angles, and water droplets also slide off by small air blow or tilting of the surface. Further, the coated sample was 10 times folded and de-folded, and it was observed that the water droplets still maintained their shape and rolled off easily. It is concluded that the superhydrophobic nature of the coating remained unaltered upon these mechanical disturbances.

### 3.3. Self-Cleaning and Anti-Fogging Properties of the Coatings

**Figure 7:**
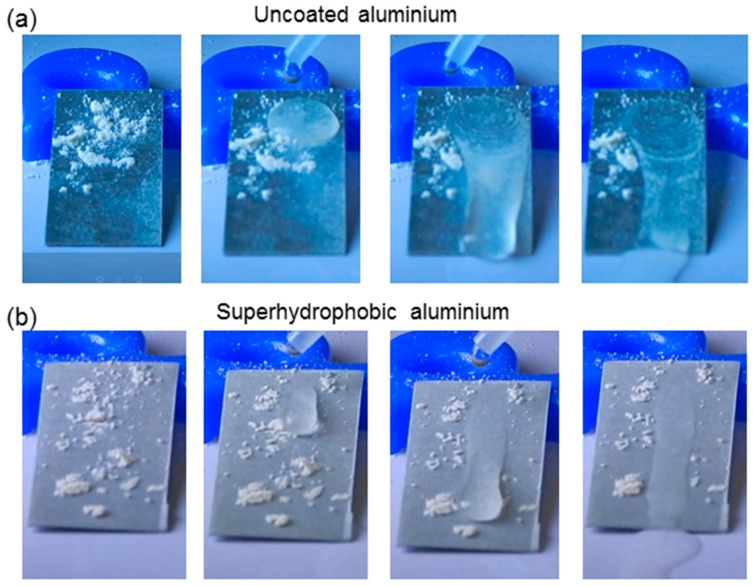
Optical images of the self-cleaning behavior of (**a**) uncoated and (**b**) superhydrophobic Al surfaces.

On superhydrophobic surfaces, liquid drops exhibit a spherical shape and low adhesion, and roll off from the surface. While rolling off, the liquid drops carry away dust particles present on the surface, this is known as a self-cleaning phenomenon [53]. In this paper, the self-cleaning property of the superhydrophobic Al surface was also studied. Chalk powder (0.5 g) of particle size 4.5 µm used as dust particles were spread on both uncoated and coated samples such that dust particles were well distributed over the surfaces. Water droplets were injected on both surfaces with the help of a needle, as shown in Figure 7. In the case of the uncoated Al sample, water immediately spread on the surface. Excessive or non-adhesive dust particles from the uncoated Al surface could be removed with the flow of water, but adhesive dust particles remained sticking on the surface (i.e., it did not show any self-cleaning property). On the other hand, for the superhydrophobic Al sample, water formed spherical drops on the coated surface, and these droplets started rolling off the surface and carried away dust particles. This implies the strong self-cleaning ability of the coating.

**Figure 8:**
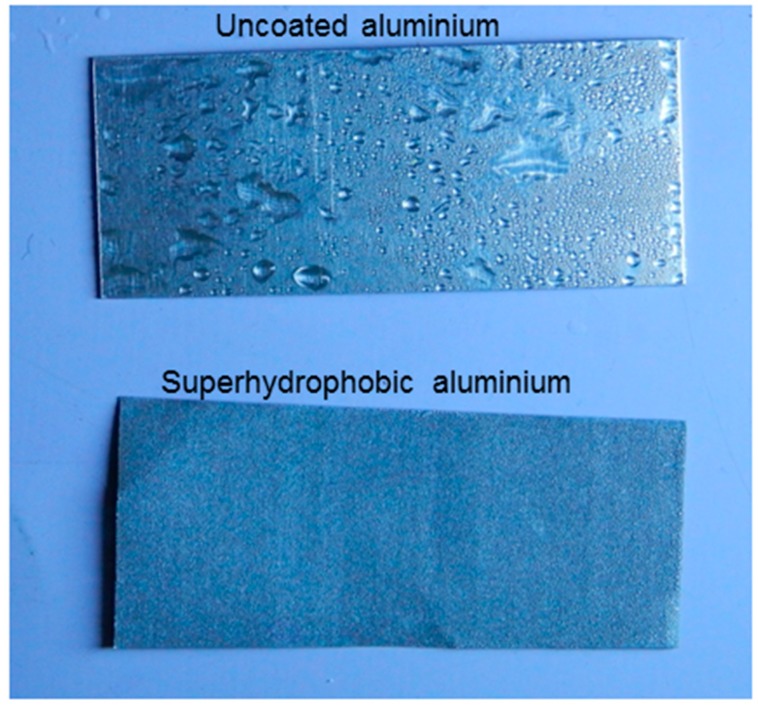
Optical images showing condensed droplets due to low temperature on uncoated and superhydrophobic Al surfaces.

In general, superhydrophobic surfaces prevent the condensation of water on its surface, known as an anti-fogging property. However, in optical windows, reduction of condensed drops could lead to a vision problem. Normally, anti-fogging is achieved by creating a superhydrophilic surface [54]. In this case, drops do not form and a water film is formed instead, but a superhydrophilic surface is more difficult to achieve. Metallic surfaces such as Al do not have vision issues; therefore, anti-fogging by superhydrophobic surfaces has tremendous applications. Recently, Zhang et al. [55] have fabricated a superhydrophobic Al surface with a controlled condensation effect. To test the anti-fogging property of the present superhydrophobic coating, both uncoated and coated samples were kept in a refrigerator for five hours and then placed in open environment. Figure 8 shows the condensate water on the surfaces. It was observed that moisture present in the air immediately condensed on the uncoated Al surface, and accumulated water droplets formed on the surface within a few minutes; whereas almost no water droplets formed on the superhydrophobic Al surface, indicating its anti-fogging property.

## 4. Conclusions

In this paper, a biomimetic Al surface that is superhydrophobic in nature was synthesized by a two-step process: creating microstructures on the Al surface by chemical etching with HCl + HNO_3_ etchant solution and then lowering the surface energy of these microstructures by chemical modification with lauric acid solution. Surface morphology, contact angle, self-cleaning, anti-fogging, and water-repellent properties were investigated under various conditions. Furthermore, the mechanical stability of this coating was also studied.

The surface morphology analysis revealed the presence of rough microstructures on the etched Al surface. After chemical modification by lauric acid, a static contact angle of 170 ± 3.9° and a sliding angle of 4 ± 0.5° were obtained. The coated sample remained floating on the water’s surface for four weeks, showing its excellent water-repellent nature. In a water jet test, the superhydrophobic surface bounced off the high-speed water stream, and no change in superhydrophobicity was found, indicating the excellent mechanical strength of the coating. The coating also withstood 100 cycles of adhesive tape peeling test, after which it displayed sticky superhydrophobicity. Additionally, mechanical disturbances due to bending and repeated folding and de-folding did not have an effect on the superhydrophobicity. Further, the coating exhibited an excellent self-cleaning property. Additionally, almost no moisture from the air accumulated on the cooled superhydrophobic surface, asserting its excellent anti-fogging property. These mechanically stable superhydrophobic Al surfaces have potential industrial applications.
